# Editorial: EGFR-TKIs for lung cancer treatment: development, application, and side effects

**DOI:** 10.3389/fonc.2025.1617788

**Published:** 2025-05-22

**Authors:** Qinglin Shen, Hexiao Tang, Shengxi Chen

**Affiliations:** ^1^ Department of Oncology, Jiangxi Provincial People’s Hospital, the First Affiliated Hospital of Nanchang Medical College, Nanchang, China; ^2^ Department of Thoracic Surgery, Zhongnan Hospital of Wuhan University, Wuhan, China; ^3^ Biodesign Center for Bioenergetics, Arizona State University, Tempe, AZ, United States

**Keywords:** EGFR-TKIs, development, side effects, application, lung cancer

## Introduction

Lung cancer remains one of the most challenging and lethal malignancies worldwide. Despite advances in early detection and prevention, non-small cell lung cancer (NSCLC), the most prevalent subtype, continues to be a clinical challenge. However, the field has been transformed by the advent of molecularly targeted therapies, particularly those aimed at the epidermal growth factor receptor (EGFR). These therapies have significantly prolonged survival and improved the quality of life for many patients. However, as this therapeutic era progresses, it brings increasing complexities, ranging from the emergence of drug resistance and treatment-related adverse effects to the shifting landscape of tumor biology.

**Figure 1:**
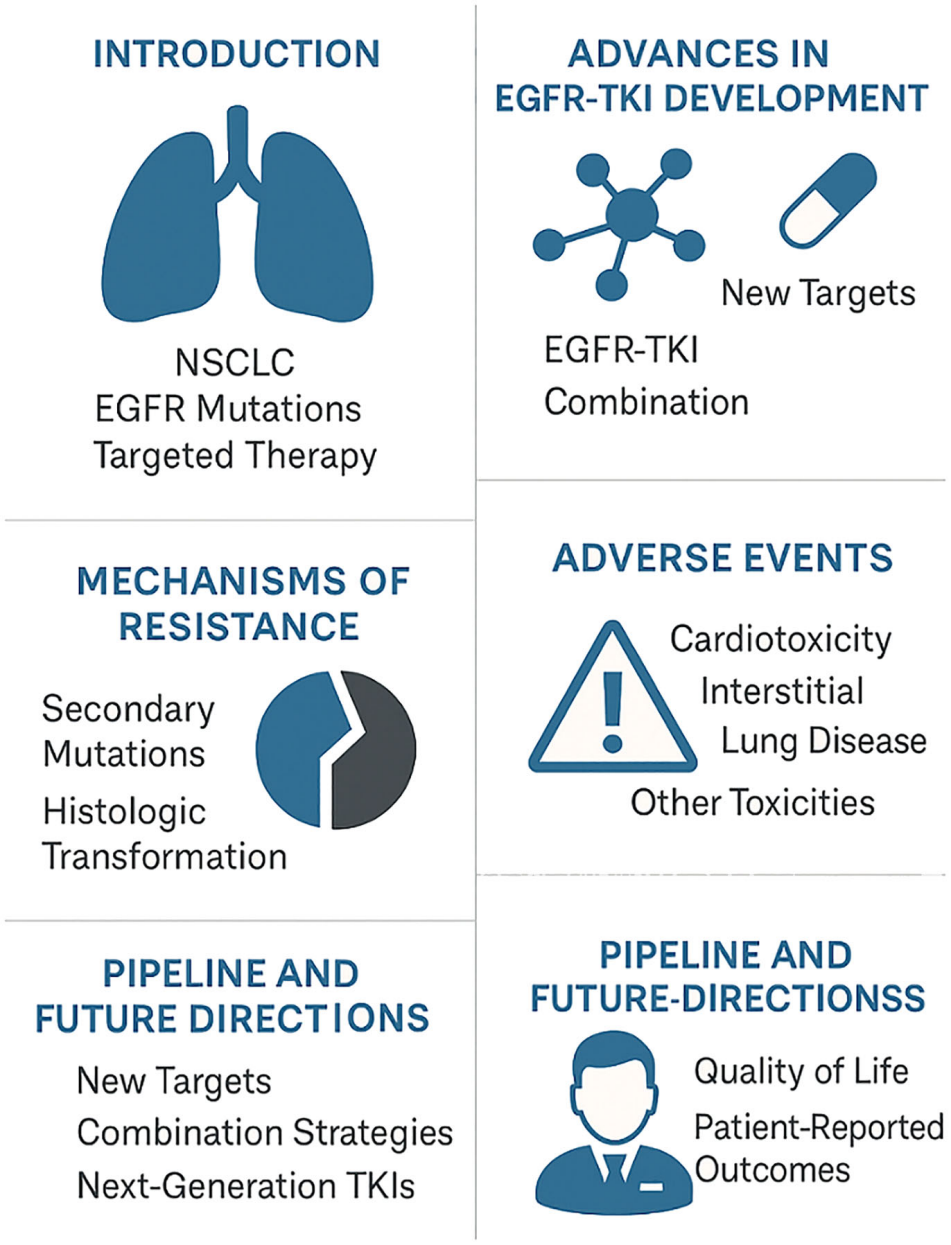
Summary of this editorial.

This editorial explores the current landscape of EGFR-targeted therapy in NSCLC, highlighting clinical progress, emerging evidence, and the critical need for innovative approaches to overcome resistance and manage adverse events (**Figure 1**).

## The advances in EGFR-TKI development

The discovery of EGFR mutations in 2004 and the subsequent development of small-molecule tyrosine kinase inhibitors (TKIs) marked a revolutionary step in lung cancer treatment (1). Osimertinib, afatinib, gefitinib, erlotinib, and newer agents such as aumolertinib and furmonertinib have redefined the standard of care for patients with EGFR-mutant NSCLC (2). These agents selectively target oncogenic drivers, disrupting cancer cell proliferation while sparing healthy tissue, thus offering a more tailored and less toxic alternative to chemotherapy.

Recent studies validate these advancements. For instance, a nationwide longitudinal study in Norway demonstrated a median overall survival (OS) of 23 months for EGFR+ patients diagnosed in recent years—a significant improvement compared to earlier cohorts (Nyen et al.). Meanwhile, aumolertinib, in addition to its efficacy, also showed remarkable improvements in patient-reported outcomes (PROs), indicating not just prolonged life, but improved day-to-day well-being (Li et al.).

## Resistance and transformation

Despite early successes, resistance to EGFR-TKIs remains almost inevitable. Tumor heterogeneity and adaptive signaling mechanisms, including secondary EGFR mutations (e.g., T790M), MET amplification, and transformation into other histologies like small cell lung cancer (SCLC), complicate the therapeutic landscape (3; 4).

Recent case reports underscore these transformations. One patient with an EGFR exon 19 deletion developed SCLC following osimertinib therapy and required a shift to etoposide and cisplatin combined with immunotherapy for disease control (Li et al.). Another case documented transformation to large cell neuroendocrine cancer (LCNEC) after almonertinib failure, emphasizing the importance of repeat biopsies to adapt treatment strategies (Cheng et al.).

Moreover, furmonertinib has shown promise in overcoming complex resistance. A single reported case yielded a progression-free survival (PFS) of 27 months in a heavily pre-treated patient with EGFR exon 20 insertion and PIK3CA mutations, supporting its potential in refractory settings (Sun and Wang).

## Adverse events: an underestimated burden

While EGFR-TKIs are generally well tolerated compared to traditional chemotherapy, accumulating data reveal a non-trivial burden of adverse events (AEs), some of which can be severe or even fatal. Osimertinib, for instance, though highly effective, has been associated with increased cardiotoxicity—including heart failure, arrhythmias, and hypertension (Wang et al.). A recent observational study found a 21.6% incidence of cardiotoxicity among osimertinib-treated patients, with smoking history, hyperlipidemia, and concurrent chemo/radiotherapy identified as significant risk factors (Wang et al.).

Network meta-analyses and pharmacovigilance reports from the FDA Adverse Event Reporting System (FAERS) further highlight drug-specific AE profiles. Afatinib and osimertinib have higher toxicity rankings, while icotinib and erlotinib are comparatively safer in terms of overall AE incidence (Shi et al.).

Perhaps most concerning are the rare but serious complications. One patient developed interstitial lung disease from almonertinib (Yang et al.), and another developed type 1 diabetes following anlotinib treatment (Chen et al.), illustrating the importance of close monitoring and personalized risk-benefit assessment.

## The pipeline and beyond: new targets and combination strategies

As resistance mechanisms continue to emerge, innovative therapeutic strategies must be developed in parallel. Whole exome sequencing (WES) has enabled the identification of rare and resistant EGFR mutations—such as G724E and K745L—that compromise drug efficacy (Nagarajan and Guda). Virtual screening against these mutations has yielded promising lead compounds, reigniting hopes for overcoming resistance at a molecular level.

Combination therapies are also gaining traction. Immune checkpoint inhibitors (ICIs), though traditionally less effective in EGFR-mutated NSCLC, have shown potential when combined with antiangiogenic agents and chemotherapy (Zhu et al.). A network meta-analysis suggests that this triplet regimen may offer the best survival outcomes, albeit with increased toxicity.

Co-targeting other HER receptors alongside EGFR represents a promising therapeutic avenue. Recently, a HER3-targeted antibody-drug conjugate, patritumab deruxtecan, received approval for the treatment of HER1-mutant non-small cell lung cancer (NSCLC) (5, 6) Moreover, targeting co-alterations such as HER2 overexpression with agents like disitamab vedotin (RC48) offers another frontier (Lan et al.). In a remarkable case, a patient with EGFR and HER2 co-alterations maintained stable disease through eight lines of therapy, culminating in disease control with RC48 and local interventions.

## Clinical implications and future directions

The current studies reaffirm the transformative power of EGFR-TKIs in lung cancer treatment. However, it also reveals a landscape fraught with complexity. Resistance is complex and often unpredictable, while adverse effects can be severe and require proactive management (Tan et al.).

Future strategies should emphasize a comprehensive approach that includes personalized treatment planning through genomic profiling and assessment of comorbidities and adverse event risks to tailor both initial and follow-up therapies. Rigorous surveillance and early detection protocols should be implemented to monitor cardiotoxicity, interstitial lung disease, and metabolic disturbances. Mechanism-driven drug development is essential, focusing on next-generation TKIs that effectively target rare mutations while offering improved safety. Additionally, exploring innovative combination regimens that integrate TKIs with immune checkpoint inhibitors and antiangiogenic agents may help delay or overcome resistance. Finally, patient-centered care should remain a cornerstone, with patient-reported outcomes incorporated into clinical decision-making to enhance both survival and quality of life.

## Conclusion

The discovery of EGFR mutations and the advent of targeted therapies have revolutionized the treatment landscape for lung cancer patients. However, as we navigate the intersection of groundbreaking innovation and growing complexity, the oncology community must stay alert and adaptive. Resistance should not be seen as a barrier, but rather as a catalyst for deeper scientific exploration and therapeutic refinement. With the continued advancement of precision medicine, proactive monitoring, and robust translational research, there is a real opportunity to transform targeted therapy from a temporary solution into a pathway toward sustained remission.
